# Organizational aspects of tissue engineering clinical translation: insights from a qualitative case study

**DOI:** 10.1186/s41231-024-00179-7

**Published:** 2024-05-30

**Authors:** Renan Gonçalves Leonel da Silva, Larry Au, Alessandro Blasimme

**Affiliations:** 1 Health Ethics and Policy Lab, Department of Health Sciences and Technology, ETH Zurich, Hottingerstrasse 10, Zurich, 8092 Switzerland; 2 Department of Sociology, Colin Powell School for Civic and Global Leadership, The City College of New York, New York, NY USA

**Keywords:** Tissue engineering, Qualitative research, Interview study, Translational research management, Research ethics, Autologous engineered nasal cartilage, First-in-human trial, Biomedical research, Health technology, Healthcare innovation

## Abstract

**Background:**

Tissue engineering is a multidisciplinary field that combines principles from cell biology, bioengineering, material sciences, medicine and surgery to create functional and viable bioproducts that can be used to repair or replace damaged or diseased tissues in the human body. The complexity of tissue engineering can affect the prospects of efficiently translating scientific discoveries in the field into scalable clinical approaches that could benefit patients. Organizational challenges may play a key role in the clinical translation of tissue engineering for the benefit of patients.

**Methods:**

To gain insight into the organizational aspects of tissue engineering that may create impediments to efficient clinical translation, we conducted a retrospective qualitative case study of one tissue engineering multi-site translational project on knee cartilage engineered tissue grafts. We collected qualitative data using a set of different methods: semi-structured interviews, documentary research and audio-visual content analysis.

**Results:**

Our study identified various challenges associated to first-in-human trials in tissue engineering particularly related to: logistics and communication; research participant recruitment; clinician and medical student participation; study management; and regulation.

**Conclusions:**

While not directly generalizable to other types of advanced therapies or to regenerative medicine in general, our results offer valuable insights into organizational barriers that may prevent efficient clinical translation in the field of tissue engineering.

## Introduction

Tissue engineering is a multidisciplinary field that combines principles from cell biology, bioengineering, material sciences, medicine and surgery to create functional and viable bioproducts that can be used to repair or replace damaged or diseased tissues in the human body [1]. Tissue engineering involves designing and fabricating artificial scaffolds, cultivating cells in vitro, and then integrating the cells and scaffolds into a functional tissue construct that can be implanted into the body. It has to potential to advance regenerative medicine, providing new treatments for a wide array of medical conditions, from organ failure to damaged tissue repair [2]. Cell sources for tissue engineering purposes include pluripotent and multipotent stem cells, progenitor cells, induced pluripotent stem cells as well as fully differentiated ones. Such cells can be sourced from donors, be retrieved in the course of biopsies, surgery and other medical procedures, or derived from embryos donated or generated for research purposes.

In the European Union, tissue engineering products are regulated by the European Medicines Agency (EMA) as advanced therapy medicinal products (ATMPs) via Regulation 1394/2007/EC [3, 4]. Between 2009 and 2022, EMA has approved only 21 ATMPs, but for 7 of them marketing authorization was either withdrawn or not renewed [5]. Of the remaining 14 ATMPs, 11 are gene therapy products, 1 is a cell therapy product and 2 are tissue engineering products, namely Holoclar (an autologous therapy to repair damaged corneal surface) and Spherox (an autologous therapy to repair knee cartilage) [6]. 

These figures show that the process of efficiently translating tissue engineering approaches to clinical use is challenging. Many factors have been identified as impediments to clinical translation including insufficient scientific knowledge, lack of dedicated funding, inadequate regulatory frameworks, ethical barriers and intellectual property roadblocks [7]. However, it is not clear what specific impediments impinge on clinical translation in the field of regenerative medicine and in tissue engineering in particular [8].

To elucidate this issue and to gain insight into organizational barriers to clinical translation in tissue engineering, we conducted a retrospective qualitative case study based on a tissue engineering multi-site translational project on knee cartilage engineered tissue grafts.

## Methods

This study aims to explore organizational impediments to the clinical translation of tissue engineering medicinal products. Given the complexity of the issue and the relative lack of published literature on such a specific topic, we decided to conduct a retrospective qualitative case study supported by a multimethod strategy of data access and analysis. The research protocol received approval from the Ethics Commission of ETH Zurich (2021-N-71).

Case study research is a kind of investigation consisting of a focused analysis of a single case aimed at shedding light on complex issues and at fostering understanding of key features of a given complex phenomenon [9]. Qualitative case studies are by their nature focused on the specific features of the case, they tend to be descriptive, and they favor heuristic interpretation over generalization of study results [10].

The choice of methods in qualitative case studies reflects researchers’ intuitions about which sources of knowledge are more likely to provide valuable insight about the case and, more in general, about the phenomenon under study [11]. Such sources can include people, documents, and ethnographic observations, to name those that are more frequently used.

Our case study is a publicly funded tissue engineering multi-site translational project on autologous cell-based engineered tissue grafts.

For the purpose of this paper, we have pseudonymized our primary data. Furthermore, we have made sure that neither participants, nor the analyzed project per se are identifiable.

This study employed a combined set of qualitative methods to analyze the practices, research behaviors, and organizational considerations in tissue engineering and translational research on engineered-cartilage implant and tissue repair, with a focus on ethical implications of development and clinical trials with ATMPs for regenerative medicine purposes.

The methods include the examination of transcripts generated from over than 200 min of interviews applied with the project’s Principal Investigator and two senior project managers (a scientific coordinator, responsible for the development of the tissue grafts, and the leader of Surgery, responsible for the replacement surgical procedure with participants); a review of twelve reports, and articles bringing project’s outcomes available on the website of the study or in the funder’s webpage, and an audiovisual content analysis of seven project presentations done by the researchers in which they explain in detail the organizational issues faced during the study.

### Interviews

Three researchers (a University Professor, a Senior Scientist and a Senior Surgeon) were selected for interviews, representing different roles within a multicentered clinical study. The interviews were conducted remotely between June and November 2021. The participants included a Principal Investigator/Professor of Tissue Engineering, a Senior Scientist/head of autologous cell-based engineered-cartilage research and development – also manager of clinical trial data and information, and a Physician/head of surgery and tissue graft implant.

The interviews aimed to gather insights into the project’s management, organizational challenges and the configuration of research processes. Data and personal information have been fully anonymized. Table 1 provides an overview of interviewees’ characteristics, including their roles within the multicentered clinical study.

**Table 1:** Participants role in the tissue engineering project (*N* = 3)

Interviewees	Affiliation	Role
Participant 1	Senior Scientist (Tissue engineer)	Project manager /Tissue Grafts Development
Participant 2	Senior physician (Surgeon)	Clinical study manager / Surgery / Cartilage replacement
Participant 3	Scientific Director	Principal Investigator / Professor / Project Director

### Documentary research

Qualified data about this case is available in official websites and sources, such as reports, project description, case presentations and publications. Aiming to complement data from interviews, and to provide data robustness through diverse materials’ sources we selected twelve documents to compose a data package to be analyzed and triangulated with data from interviews.

The package consisted of official reports, scientific publications, conference proceedings, and relevant informational pieces from clinical blogs. These documents specifically discussed practices, research behaviors, and organizational considerations related to tissue engineering and translational research on engineered-cartilage implant and tissue repair. The analysis of these documents provided a comprehensive understanding of the research field. Table 2 presents the details of the twelve selected documents, including the original research articles, reports and other project outcomes. Data about publications were fully anonymized to avoid participant identification.

**Table 2:** Selected documents (*N* = 12)

Documents	Year	Publication type	Field | Area
Doc1	2021	Research article	Tissue engineering
Doc2	2014	Research Article	Medicine
Doc3	2019	Book chapter	Translational research / Clinical Trial
Doc4	2022	Project Presentation	Tissue engineering
Doc5	2020	Periodic Project Report	Clinical trials
Doc6	2016	Review Article	Translational research
Doc7	2021	Press release	Tissue Engineering Project
Doc8	2019	Original Article	Translational research
Doc9	2017	Review Article	Medicine
Doc10	2019	Book Chapter	Medicine
Doc11	2020	Project Presentation	Cell Biology / Translational Medicine
Doc12	2022	Project Presentation	Tissue engineering

### Audiovisual content analysis

Much evidence about the organizational challenges and emerging questions associated with first-in-human clinical trials in tissue engineering are available online in the format of videos and audiovisual content. The use of materials available digitally has gained space in multimethod qualitative studies that have recently introduced audiovisual content analysis as a mechanism to access reliable information about cases that deal with a scarce amount of data due to its novelty, restricted access or because it is a field under development [12].

Seven recorded videos (publicly available) were examined as part of the audiovisual content analysis. These videos were uploaded to the official channels of the project and funders and were fully available on streaming platforms. The audiovisual content analysis was fundamental in gaining insights into the project presentations, highlighting key aspects of the research, methodologies, and outcomes. The analysis of these materials complemented the findings from the interviews and documentary research.

To ensure confidentiality and protect the privacy of the individuals involved in the research, all information related to the researchers’ identity, institutional affiliation, and funders was anonymized. This anonymization process was in accordance with the consent forms signed by the participants and agreed upon by all parties involved. Table 3 outlines the characteristics of the seven presentations analyzed in the audiovisual content analysis, including the video source and key themes addressed.

**Table 3:** Selected Audiovisual Content (*N* = 7)

Audiovisual content	Year	Type	Field | Area
AvC1	2022	Academic event or scientific conference	Multidisciplinary
AvC2	2022	Science Report	Tissue Engineering
AvC3	2017	Short video extracted from press release	Tissue Engineering
AvC4	2018	Short video extracted from press release	Medicine
AvC5	2018	Project presentation	Tissue Engineering
AvC6	2016	Science Report	Medicine
AvC7	2015	Academic event or Scientific conference	Medicine

The combination of these qualitative methods provided a multiperspective analysis of the tissue engineering clinical translation, with a specific focus on organizational aspects. While not directly generalizable to other types of advanced therapies or to regenerative medicine in general, our results offer valuable insights into organizational barriers that may prevent efficient clinical translation in the field of tissue engineering.

Confounding variables and missing data are crucial considerations in our study. Although we did not address confounding variables, they could significantly impact the scope and conclusions of our analysis. Factors such as the geographical locations of participants and research/clinical staff, the level of technological novelty in first-in-human tissue engineering clinical translation (which may differ from other similar translational agendas), and contextual considerations within the research project, including the expertise of the team of scientists and clinicians, and years of experience in tissue engineering, are examples. We did not access data on these potential variables because our study aims to provide a broader overview of challenges and how stakeholders approach solutions.

## Results

Our study identified various challenges associated with first-in-human trials in tissue engineering, particularly related to five themes: (1) logistics and communication, (2) research participant recruitment, (3) clinicians and medical student participation, (4) study management, and (5) regulation.

### Logistics and communication

Logistics and communicational issues emerged as a common concern between researchers and clinic stakeholders. It was noted that the coordination and effective exchange of information among various parties involved in the research process posed challenges.

For a participant leading the management of clinical trial’s data for over than a decade, problems derived from persistent difficulties in coordinating time and availability of both scientists and surgeons that advance the clinical trial in parallel with their current professional activities and commitments.

None of us have this trial as our exclusive activity or work responsibility. We all do it in parallel with teaching, supervising students, other projects and publishing results from previous projects. And the management of such a study takes a lot of time. To me, part of the challenges relate to this issue, and would be great to have a research team dedicated full-time to the trial, but this is not possible (Participant 2).

In this trial, time was directly related to regulations to bring results from the lab to the bedside, and with communication issues among scientists and healthcare professionals from different fields of expertise. A participant stated that time is a relevant variable in clinical trials in tissue engineering, and that to implement the study in accordance with all the rules and protocols takes long that demands better strategies of time allocation and mechanisms to push trials as routine of clinical practice.

Things take a lot of time in this kind of [clinical] study (…) Just to show you, for instance, we start this research back in 2000 and the first applicable standard operating procedure (SOP) for GMP production was examined in 2010.” (AvC 1).

I think it’s important to start as complicated a necessary to understand the system, to design it. But then simplify it. Because otherwise it will not make it into the routine clinical practice (Participant 3).

The problem of time expenditure in covering regulatory demands was reinforced by a researcher during a presentation in 2022, framed as “challenges associated with moving lab results to translational studies”. They explained the logistics needed to set the laboratory to run such a first in human trial.

We went from a research lab to clinical studies. So, we had to move our lab to translational studies. And for that, you need to change your research protocol. You have to go for GMP (Good manufacturing parties) production and GMP compliant reagents, so, a lot of things to change in the end. To be compliant with this, we needed a quality management system composed by plenty of documentation… In our group we do both product manufacturing following GMP and also to deal with the paperwork for the clinical studies together with the surgeons, like study protocols, inform consent, etc. all this things… (AvC3).

The problem of logistics is also seen as result of the implementation of best practices and standards, which require acts of interpretation to fit local conditions and to guarantee that protocols will be respected accordingly by a team of researchers and clinicians spread in multiple countries:

We have a full team which is working on this. Because yes, there are some documentation that gives the guidelines. These are huge packs of documentations which are not simple to decipher and to interpret. So it requires experts. And I can say only with research culture, I would have trouble to address these issues… We cannot trust that a scientist will just read an article and understand what necessary. (Participant 2)

The communication issue was framed by a clinical study manager as the challenge “to make people to speak the same language” (Doc4). Researchers expressed the need for improved communication protocols to facilitate smooth collaboration and streamline the progress of the trial. A participant said it demands a clear communication between styles of thinking from science, engineering and clinics. To translate engineering principles into clinical practice, for this team member, entailed the need to become more scientific, and to master the most up-to-date scientific advances in a particular field.

For me it’s really dedication of being like a scientific surgeon. And I narrowed my clinical field to be more specialized, and to be able to cover all (AvC4).

### Research participant recruitment

Effective management of participant recruitment also appeared as a notable organizational challenge in first-in-human trials of tissue engineering, requiring improved strategies and procedures. One interviewee stressed the relevance of being clear about safety to effectively recruit research participants.

In patient recruitment, a crucial issue of the clinical study is to demonstrate safety and efficacy, which should be given special attention already during the planning of the trial. (Doc3)

Skepticism among patients and their preference for similar tissue grafts already available on the market were identified as significant challenges in candidate selection. Researchers found that patients often had reservations and hesitations towards participating in trials involving novel tissue grafts.

(…) some people would not take the risk to get something new. They say, I go for what is already on the market (…) they want the new treatment instead of a prosthesis (…) I mean people think twice about whether they want to try something new or if they go for what is already available in the market for 10 years. (Participant 2).

Tissue grafts and biomaterials’ manufacturers work can, sometimes, clash with clinical priorities. From the point of view of tissue engineers, producing a tissue graft takes time, and many problems might emerge in this process. However, on the other hand, an interviewee points out that clinical workload (time) and lack of scientific skills affect recruitment’s efficiency due to technical issues placed beyond the clinical expertise.

The main problem is clinical workload. So time for research. I would also say it is related to the lack of research skills. There’s a lack of scientific knowledge on those methods. And also not only the scientific methods but creativity, ideas or innovation (Participant 3).

Transparency issues pertaining to participant recruitment and the communication of risks associated with first-in-human trials were identified as crucial concerns by researchers.

De facto, when they have for example a patient that does not entirely qualify for the inclusion criteria let’s say, then the tendency would be, well ‘why don’t we change the inclusion criteria so that we fit this patient’. And I must say that it never reaches a clash because we say, ‘well no’. Because otherwise we cannot address with the same powered design in this clinical trial with this scientific question. And so they accept it. But the tendency would be again to introduce always this level of flexibility (Participant 2).

The challenge of participant recruitment also relates to issues of transparency among groups of researchers working in the study. For a PI of the study, it is hard to control all variables emerging at the clinical side in multi-centered trials due to different approaches adopted by researcher to recruit participants.

I feel privileged to work with illuminated surgeons and clinicians. But from what I see in the international context, some [clinicians] offer an experimental procedure to a patient as a praxis [participant meant without fully discuss its risks and implications]. So new [protocols] have to be developed, have to be introduced in the clinic, but in the context of well-designed and transparently communicated trials. (Participant 1).

### Clinician and medical student participation

The participation of clinicians and medical students in first-in-human trials was observed to be lower than initially anticipated by principal investigators (PIs) and project managers.

Clinician-researchers often take on multiple roles in translational research studies. Other than relying on themselves for recruitment, the team member also goes on to explain that they also rely on *relationships and networks* of co-workers, peers, and potential collaborators to recruit patients:

I have my outpatient service. So I see lots of patients myself. But if something is launched I inform colleagues to also watch out for possible patients. And we have a certain program of research education in the hospital. And every now and then I present, and then I also mention the ongoing projects so that people are informed. And it’s more or less the same in the lab. So we have progress reports, and where we have meetings. And maybe, sometimes it’s also just when having a coffee that you talk about such things. So very informal sometimes” (Participant 3).

Another researcher-clinician on the team also reports the importance of informal ties: “For instance [our team leader], if he know some surgeon who are interested in other clinic, it often starts like this. If you know someone, because then you know if people are motivated, if they work seriously, and everything. It’s a bit easier” (Participant 1). Informal ties thus not only help recruit potential patients, but also helps identify suitable collaborators.

Despite efforts to engage medical professionals in these trials, their involvement was below the expected levels. This limited participation raised concerns regarding the overall effectiveness and feasibility of the trials, as well as the potential impact on data collection and analysis. Then, to facilitate translational research, hospitals and universities can also implement *organizational strategies* of proximity and relation-work to facilitate exchanges in knowledge and ideas. As an interviewee responded:

Lots of surgeons were going into the lab for research year to learn basic science, to understand what is happening there, and also maybe giving back with this knowledge into the clinic. So there’s was a quite intense exchange. This is quite was quite key. And this was also supported by the department that you get the position and that you get a salary. Which is not the standard as a clinician, that you’re going to the lab and you receive a salary… So I was supported and was with overarching structure of the surgery and the university” (Participant 2).

### Study management

As a multidisciplinary team engaged in the translation of engineering principles to the clinical context, there were moments when respondents highlighted potentials for clashes and conflicts in priorities, goals, and approaches between different team members.

If we want this science translated to a clinical setting, the challenge will be in my view to streamline and in most cases simplify processes to make them practical. I think some of the approaches that are being pursued are fantastic, are just conceptually so sophisticated and advanced. But the possibility to implement them into simple protocols that can be adopted by manufacturing groups and that can be transferable into the clinic is the main challenge (Participant 1).

The standardization of routines, availability of surgeons, the non-rare change of protocols for first-in-human trials in tissue engineering, and the management of patient participation were highlighted as key issues of the study management by PIs. A surgeon member of the clinical study pointed out the implementation of a standardized routine as a critical collective practice for the success of a clinical trial in tissue engineering, once it improves the reliability of procedures from the lab to the surgical table.

This is the requirement for scientific advances to be introduced clinically: a standardized routine. Because if we do not understand systems, we cannot control them. And people used to say, that the best way of understanding something is to is to create it. Because then you have a grasp on it (…) Standardization and reliability are necessary for clinical trials [and] for entering the routine practice for certain therapies (Participant 3).

A participant notes the need for standardization across organizations involved in the trial:

For this large multicenter study we are the ones who provide all the documentation to all the other centers. For instance, we write patient information, all those things, and they are translated to other languages if necessary of course. We have a standard operating procedure, so protocols, and try to make sure that everyone is following the same protocol in each country. We kind of centralize all the information when they send it back as well, if patients had problems, adverse events, everything… (Participant 2).

While strategies of proximity and relation-work may help with local regulators, regulators further away may be yet another challenge that researchers face when attempting to seek approval for their new projects.

### Regulation

Restrictive regulations due to the absence of protocols, limited patient availability, and translational lag in certain national contexts were identified as factors that compelled researchers to seek collaborations with international partners, as mentioned in a study report “At the same time, regulatory issues have become more complex, and there is no clear road map.” (Doc5).

The absence of established protocols specific to first-in-human trials in tissue engineering created uncertainty and hindered the overall efficiency of the research process. Since protocols in this area are constantly in change, it was framed by a scientist as a key challenge involved in the management of the clinical study.

You always need to continuous the development of your product, because clinical indications of our products are constantly changing. For example, sometimes the patients have larger defects to be treated. Then, we need to provide larger (tissue) grafts, so we need more cells. At the beginning we use to culture our cells with blood from the patient, so, no foreign product, so then we realized we would need to much blood from the patient to go for a larger production (laughs), so, not really nice as well… So then we changed products, for instance. Then, for that, you have to validate changing in raw material (replace autologous serum by hPL) and do a comparability study to show it is going to be the same.” (AvC1).

But the relationship between research teams with regulatory authorities also differs depending on the locale. As the project leader explained:

“We have received a lot of support and a lot of signals of flexibility to help us enter the early phases of the clinical trial. Clearly from a pilot trial, you want to reach marketing authorization for a product, then everything becomes more stringent. But in academic settings, to have an investigatory initiated clinical trial in [named the country], we have found the trajectory, the pathway is indeed facilitated by the regulatory agency. Which is less at the [international] level, and absolutely not the case at the [named third country] level… So we can consider ourselves privileged” (Participant 1).

According to an article cited by an interviewee (Doc9), the regulatory issues among scientists and physicians in multi-centered trials are directly associated to the lack of harmonization of regulations in the field internationally.

“A challenge for academia can arise not only from the regulations themselves, but also, in multi-centric studies, from the lack of harmonization between different countries. This becomes apparent in the different interpretation of European regulations, different implementation of directives in the national law of each country, requirements for qualification of personnel as well as in requirements for the manufacturing processes regarding quality of reagents and testing. This may lead to acceptance of a clinical trial in one country, but not in another, requiring several submissions until all authorities are satisfied.” (Doc9).

Due to regulatory constraints and inadequate patient pool in some countries, researchers faced difficulties in conducting the trials solely within national boundaries. As a result, international collaborations became imperative for overcoming these challenges and ensuring the progress and success of the research (Doc6 and AvC3).

Aside from external organizations, proximity and relation-work also aides in the management of regulatory oversight and scrutiny of research. As the surgical research member points out:

I recommend to contact the authorities early and keep in touch with them. (…) For research groups [that] have no experience with regulators, I tell to contact or collaborate with groups with experience on this. Those might have some ideas about good platforms for translation, that helps with regulation because it is quite a big field and takes a lot of work and time. And also lots of money. It is difficult for a small research lab to cover this (Participant 2).

## Discussion

Tissue engineering offers great prospects in the field of regenerative medicine to heal and repair tissue and bodily structures damaged by injury or disease. The clinical advantage of tissue engineering is that it offers biocompatible solutions that can be customized to the specific needs of patients, by harnessing the natural healing capacities of the human body while sustaining such process through bio-engineered scaffolds that greatly enhance the prospects of healing damage. Nevertheless, due to its technical complexity, tissue engineering poses critical challenges that demand a holistic approach to organizational management. Some organizational issues inherent to first-in-human clinical trials have been documented in the literature, especially regarding problems associated to market authorization and the regulatory processes [13].

In our study, researchers have also pointed persistent challenges of tissue engineering trials, as those also identified in our empirical analysis regarding to study management, clinician and medical student participation, recruitment and transparency. We highlight roles played by expertise access, resilient public engagement and efficient dialogue with patients, translational education and training, routines, and early consideration to ethics and regulation of new technologies as potential strategies to address challenges in tissue engineering trials. In Table 4 we summarize key challenges and its corresponding solutions as highlighted by participants of the study.

**Table 4:** Tissue engineering clinical translation challenges and potential solutions highlighted by the participants of the study

	Challenges	Solutions
**Logistics and communication**	• Persistent difficulties in coordinating time and availability of both scientists and surgeons • Interpretation to fit local conditions to guarantee that protocols are respected • Communicational issues due to different styles of thinking from science, engineering and clinics	• Expertise access • Organizational change
**Research participant recruitment**	• Demonstration and communication of safety and efficacy of new technologies • Patient preference for similar tissue grafts already available in the market • Clinical workload and lack of scientific skills affect recruitment’s efficiency due to technical issues placed beyond the clinical expertise	• Resilient public engagement • Efficient dialogue with patients (transparency) • Organizational change
**Clinician and medical student participation**	• The participation of clinicians and medical students in first-in-human trials was observed to be lower than initially anticipated by principal investigators (PIs) and project managers	• Translational education and training
**Study management**	• Need for standardization of routines across organizations • Availability of surgeons, • Non-rare change of protocols for first-in-human trials in tissue engineering, • Management of patient participation • Time allocation and mechanisms to push trials as routine of clinical practice • Potential clashes and conflicts in priorities, goals, and approaches between different team members	• Routines • Organizational change
**Regulation**	• Restrictive regulations due to the absence of protocols, limited patient availability, and translational lag • Time expenditure in covering regulatory demands • Collaboration among research teams and regulatory authorities	• Early considerations to ethics and regulation

Source: elaborated by authors

A recurrent theme discussed by the literature on translational research’s management deals with the importance of “expertise access” in such trials. The involvement of diverse specialists, including clinical researchers, cell biologists, surgeons and other healthcare professionals is integral to comprehensively assess the safety and potential efficacy of novel compounds. Wilkinson et al. (2017) [14] highlight the significance of early engagement of cross-disciplinary teams to facilitate robust trial design and ensure efficient execution. Similarly, Jones and Smith (2019) access to specialized expertise is important in identifying and addressing potential risks, thereby safeguarding the well-being of trial participants [15].

Simultaneously, the literature shows the critical role of organizational change in overcoming the challenges posed by first-in-human trials. Klim et al. (2020) advocate for the establishment of dedicated translational research units that facilitate streamlined decision-making processes and foster collaboration among stakeholders [16]. Such organizational innovations encompass adaptive trial designs, which allow flexibility in protocols based on emerging data, leading to more efficient resource allocation (time and expertise included), and quicker identification of compound attributes. Additionally, organizations should consider more efficient dialogue with patients as part of institutional mechanisms to both facilitate communication of benefits of new biotechnologies and to enhance access to future study participants.

The implications of organizational innovation extend beyond trial execution and influence the broader landscape of healthcare innovation. As noted by Brown et al. (2021), effective collaboration facilitates more accurate translation of preclinical data, reducing the likelihood of trial failures and optimizing resource allocation [17]. Innovative organizational approaches can expedite trial timelines and contribute to cost savings [18].

Overcoming logistical barriers, such as the availability of specialized professionals, can be particularly challenging in emerging areas of therapeutic intervention such new bioengineered tissue grafts and other biotechnologies holding higher levels of risk and translational failure [19]. Addressing these challenges is essential to fully realize the potential benefits a translational interface between science, engineering and medicine in clinical trials.

Finally, early ethical and regulatory considerations are a central component of first-in-human trials. Ensuring access to relevant expertise aligns with the ethical obligation to minimize risks to participants [20]. Furthermore, organizational innovations contribute to transparency and participants’ autonomy through enhanced communication, enabling participants to make better informed decisions about their involvement in trials. This ethical dimension adds weight to the relevance of both expertise access and organizational change in first in human trials.

Our study has limitations. The small number of participants might lead to questions about the statistical significance and relevance of the data used to formulate our hypotheses and considerations regarding the challenges and solutions to improve tissue engineering clinical translation. However, the limited number of research and clinical staff in the field has been highlighted by interviewees as a significant challenge within this realm of research and development. Additional limitations could arise from the anonymization of study participants, which may obscure potentially pertinent information concerning technical and organizational aspects related to the clinical translation of a specific biotechnology.

## Conclusions

Our study illustrates the intricacy of organizational challenges in first-in-human clinical trials in tissue engineering and highlights the pivotal roles played by study management and interdisciplinary expertise to accommodate translational research competences and new knowledge. By harnessing specialized expertise and embracing innovative trial design approaches, stakeholders can navigate the complexity of clinical translation in tissue engineering more effectively, ultimately contributing to improved tissue engineering clinical translation.

These findings shed light on the complexities needed to conduct first-in-human trials in tissue engineering and underscore the need for effective strategies, standardized protocols, and international collaborations to overcome these challenges and advance the field.

While not directly generalizable to other types of advanced therapies or to regenerative medicine in general, our results offer valuable insights into organizational barriers that may prevent efficient clinical translation in the field of tissue engineering. The processes of clinical translation in tissue engineering have significantly advanced over the last decade.

Enhancing organizational tools, refining funding mechanisms, and incentivizing early ethical and regulatory scrutiny of new biotechnologies to tackle the issues outlined in our study could significantly reduce translational lag and prevent delays in promising tissue engineering clinical translation.

## Data Availability

The datasets used and/or analysed during the current study are available from the corresponding author on reasonable request.
